# A rare cause of myocardial infarction and ventricular tachycardia in a young male with HIV/AIDS - spontaneous coronary artery dissection: A case report

**DOI:** 10.34172/jcvtr.2021.24

**Published:** 2021-04-06

**Authors:** Krishna Prasad, Tanushi Aggarwal, Prashant Panda, Ganesh Kasinadhuni, Yash Paul Sharma

**Affiliations:** ^1^Department of Cardiology, Post Graduate Institute of Medical Education and Research, Chandigarh, India; ^2^Department of Internal Medicine, Post Graduate Institute of Medical Education and Research, Chandigarh, India

**Keywords:** Spontaneous Coronary Artery Dissection, Ventricular Tachycardia, Human Immunodeficiency Virus, Acquired Immunodeficiency Syndrome

## Abstract

HIV/AIDS is a multisystemic disorder and occurrence of cardiovascular disease is higher compared to non-HIV individuals. Spontaneous coronary artery dissection (SCAD) remains a rare and underdiagnosed cause of acute coronary syndrome (ACS), even in modern day era. SCAD is predominantly seen in young to middle aged females and present as a non-atherosclerotic cause of myocardial ischaemia, infarction or sudden cardiac death (SCD); with or without ventricular arrythmias. Ventricular tachycardia (VT) can sometimes be the initial presentation of SCAD. HIV associated arteriopathy can predispose to occurrence of SCAD. We report a case of a 38-year-old male suffering from HIV/AIDS, with no conventional risk factors presenting as VT. Coronary angiogram showed SCAD in right coronary artery without any flow limitation.

## Introduction

Spontaneous coronary artery dissection (SCAD) is a rare nonatherosclerotic cause of myocardial infarction (MI) in young and middle aged population, predominantly seen in peripartum females. It remains a largely undiagnosed cause of myocardial ischemia, partly because of lack of angiographic recognition. The etiology of SCAD has been associated with steroids, which can explain the higher incidence in peri partum period with higher fluctuating levels of various steroidal hormones. The etiology of SCAD has also been linked to fibromuscular dysplasia (FMD) where the hematomas in the vessels can predispose to SCAD. SCAD can present as a non-atherosclerotic cause of myocardial ischaemia, infarction or sudden cardiac death (SCD); with or without arrythmias. HIV/AIDS is a multisystemic disorder. Cardiac involvement is predominantly in the form of coronary artery disease, however pericarditis, myocarditis, heart failure, HIV associated cardiomyopathy, tumors can also occur.^1,2^ HIV associated arteriopathy can cause coronary artery dissection presenting as acute coronary syndrome. We present a case of 38-year-old gentleman with HIV/AIDS, who presented with recurrent episodes of sustained ventricular tachycardia (VT) and coronary angiogram showing spontaneous coronary artery dissection.

## Case Presentation

The case was known as a 38-year-old gentleman with HIV/AIDS on combination highly active antiretroviral therapy (HAART) (Tenofovir, Lamivudine, Efavirenz), with recurrent episodes of palpitations.

**Figure 1:**
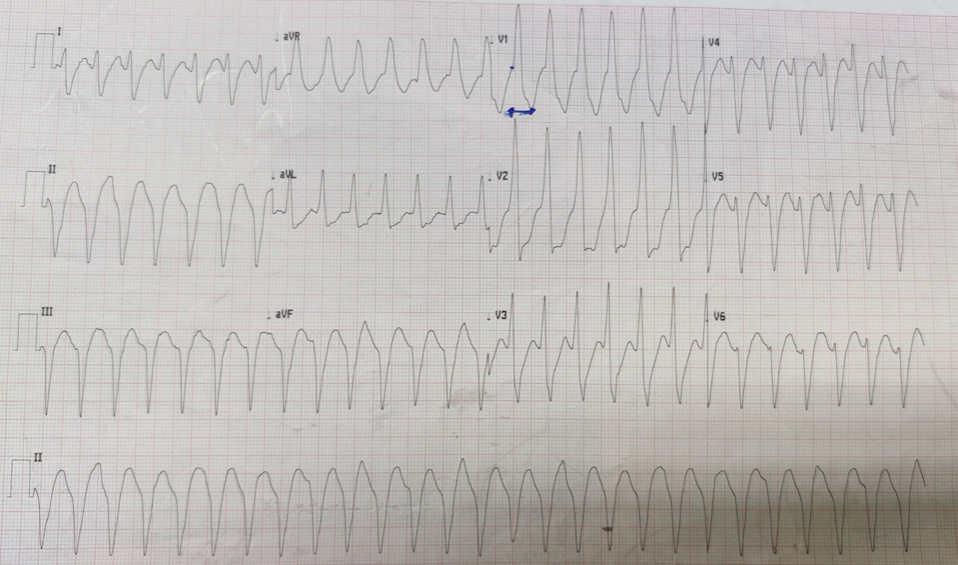
Electrocardiogram showing wide complex tachycardia with RBBB morphology with superior axis suggestive of monomorphic VT of LV origin

**Figure 2:**
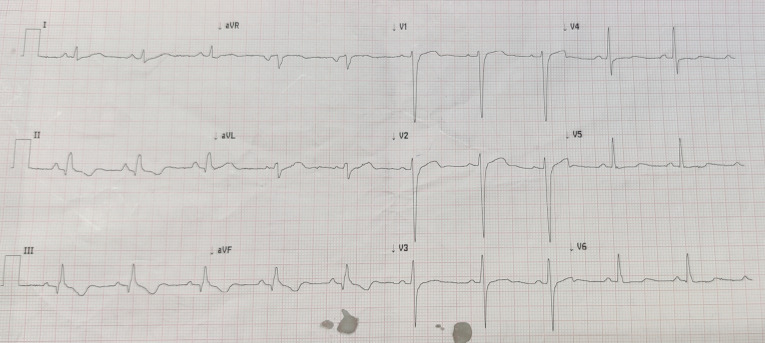
Electrocardiogram showing Sinus rhythm with Q waves and T wave inversions in Inferior leads

**Figure 3:**
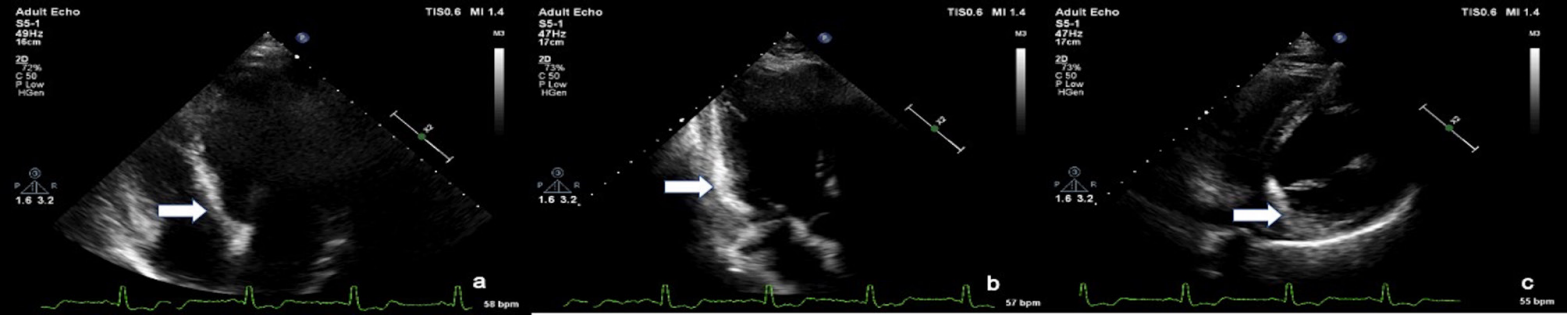
Echocardiogram images in a Apical 4 chamber view, b. Apical 3 chamber view, c. parasternal short axis view showing increased echogenicity of myocardium in the RCA territory (arrow)

**Figure 4:**
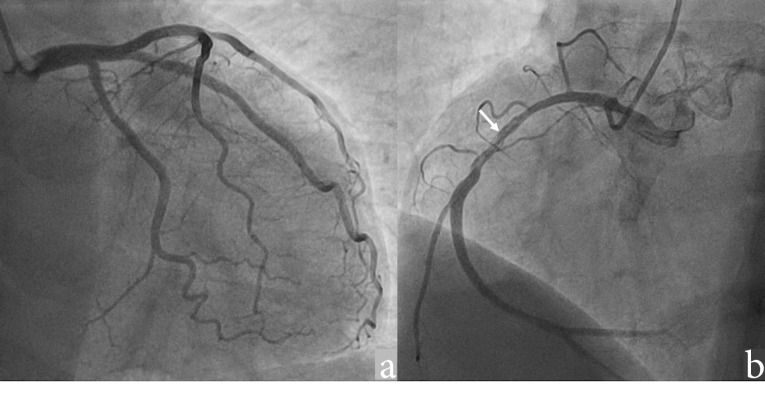
Angiogram images showing (a) Normal left coronary artery (b) Spontaneous coronary artery dissection in the mid RCA

The patient had no history of hypertension, diabetes, smoking, or dyslipidemia. He denied any history of angina or syncope. On examination, the patient was having tachycardia and a blood pressure of 80/50 mm Hg with diaphoresis. During the episode of tachycardia ECG revealed monomorphic VT (right bundle branch block morphology with superior axis) (as shown in Figure 1). Sinus rhythm was immediately achieved by direct current cardioversion. Electrocardiogram (ECG) during sinus rhythm showed q waves in inferior leads with T inversion and QTc interval of 420 ms (as shown in Figure 2) suggestive of evolved inferior wall MI. Blood investigations including complete blood count, electrolytes including potassium, calcium magnesium, liver and renal function tests, and the lipid profile were within normal limits. Troponin T was elevated at 4.3 ng/mL. CD4 cell count of the patient was 987/mm^3^. Echocardiography of the patient showed hypokinesis in right coronary artery (RCA) territory with an EF of 50 % (as shown in Figure 3, Supplementry File 1, Video S1). He was treated with amiodarone infusion, dual antiplatelets (aspirin and clopidogrel), atorvastatin, metoprolol and low molecular weight heparin. The patient underwent coronary angiography which revealed spontaneous coronary artery dissection in mid RCA (as shown in Figure 4, Supplementry File 2, Video S2). Rest of the coronary vessels were normal (Supplementry File 3, Video S3). In view of lack of flow limiting stenosis and TIMI III flow, revascularization was not done. For secondary prevention, implantable cardiac defibrillator has been advised for the patient, but the patient has refused the same due to financial reasons. The patient developed no further episodes of VT during hospital admission and on follow up after a month.

## Discussion

HIV/AIDS is a multisystemic disorder and affected individuals have an increased incidence of cardiovascular events (myocardial infarction and stroke) compared to non-HIV individuals.^3^ Cardiac involvement can be in the form of pericarditis, endocarditis, myocarditis, cardiomyopathy, heart failure, arrhythmias in addition to the coronary artery disease.^2^ The mechanism underlying the increased risk of acute coronary syndrome in these patients is debatable involving many factors like inflammation, CD4 count depletion, hypercoagulability, endothelial dysfunction, dyslipidemia, metabolic derangement caused by HAART, impaired arterial elasticity etc.^3,4^ Cardiac arrhythmias have been reported in patients with HIV/AIDS and are predominantly due to the QT prolongation by HAART and other drugs like pentamidine, pyrimethamine, and cotrimoxazole. Other reasons for arrhythmias include myocarditis and acute coronary syndrome.^5^ SCAD as a cause of arrhythmias have been reported rarely in patients of HIV/AIDS. SCAD occurs due to arteriopathy predisposing to intramural hematoma. Thus HIV vasculitis or related arteriopathy can be a causative factor. We could find only one case report describing the occurrence of SCAD in a young female with HIV.^6^ Another report of stroke in HIV due to right vertebral artery dissection has been described.^7^ Arteriopathy in HIV can occur due to multiple reasons like HIV related vasculopathy, anti-retro viral therapy etc. Whether there is an interplay between HIV associated inflammation and arteriopathy leading to SCAD is difficult to establish but such a possibility should be considered.

SCAD, first described in 1931 by Pretty et al is an infrequent cause of ACS caused by an intramural hematoma separating intima-media complex from the underlying vessel and compressing the true lumen causing ischemia/infarction. The actual number of cases may be higher due to lack of angiographic or intravascular imaging recognition, lack of standard criteria and low reporting.^8^ In single centre studies the prevalence may be as low as 0.78% to as high as 4% of the ACS cases.^8,9^ It is predominantly a disease of young to middle aged females with a female to male ratio of 2:1. Women constitute even higher percentage (87% to 95 %) of the cases in some series.^10^ Most of the cases of SCAD are precipitated by physical (predominantly in males) and emotional factors (predominantly in females).^11^ However our patient has not given any history that would have precipitated SCAD. The most common site involved in SCAD is left anterior descending artery (which accounts for 60% of the cases) followed by RCA (which is more commonly seen in males).^12^ Our patient is also a male patient with SCAD in mid RCA.

SCAD have varied presentations ranging from asymptomatic individuals to various life threatening events like myocardial ischaemia, myocardial infarction or SCD; with or without arrhythmias.

Coronary angiography is the investigation of choice and identifies most of the cases of SCAD. In cases of uncertainty intravascular imaging like optical coherence tomography helps in differentiating SCAD from recanalized thrombus or woven coronary artery. Intravascular ultrasound because of low spatial resolution is less helpful. CT coronary angiogram because of its lower spatial resolution for smaller vessels, motion artefacts and infrequently identified dissection is of not much use.^10^

## Conclusion

In our patient HIV/AIDS could have been the causative factor for SCAD, previous myocardial infarction and ventricular tachycardia in this patient because the patient is a young male without any traditional risk factors like diabetes, hypertension, smoking or family history of premature coronary artery disease, although such an association was not conclusively established.

## Acknowledgements

None.

## Funding

No funding has been taken for the study.

## Ethical approval

Ethics committee approval was not sought since all the treatment was according to acceptable norms.

Written and informed consent has been taken from the patient and the same is available with the authors.

## Competing interest

The authors declare no competing interests.

## Supplementary Files


supplementary file Video S1.

supplementary file Video S2.

supplementary file Video S3.
